# Quantitative Magnetization EXchange MRI Measurement of Liver Fibrosis Model in Rodents

**DOI:** 10.1002/jmri.28228

**Published:** 2022-05-06

**Authors:** Ella Wilczynski, Efrat Sasson, Uzi Eliav, Gil Navon, Uri Nevo

**Affiliations:** ^1^ Department of Biomedical Engineering, Faculty of Engineering Tel Aviv University Tel Aviv Israel; ^2^ School of Chemistry, Faculty of Exact Sciences Tel Aviv University Tel Aviv Israel; ^3^ Sagol School of Neuroscience, Faculty of Life Sciences Tel Aviv University Tel Aviv Israel

**Keywords:** MEX MRI, liver fibrosis, animal model, collagen fraction

## Abstract

**Background:**

Quantitative MRI can elucidate the complex microstructural changes in liver disease. The Magnetization EXchange (MEX) method estimates macromolecular fraction, such as collagen, and can potentially aid in this task.

**Hypothesis:**

MEX sequence, and its derived quantitative macromolecular fraction, should correlate with collagen deposition in rodents liver fibrosis model.

**Study Type:**

Prospective.

**Animal Model:**

Sixteen adults Sprague–Dawley rats and 13 adults C57BL/6 strain mice given carbon tetrachloride (CCl_4_) twice weekly for 6 or 8 weeks.

**Field Strength/Sequence:**

A 7 T scanner. MEX sequence (selective suppression and magnetization exchange), spin‐echo and gradient‐echo scans.

**Assessment:**

Macromolecular fraction (*F*) and T_1_ were extracted for each voxel and for livers' regions of interest, additional to calculating the percentage of *F* > 0.1 pixels in *F* maps (high‐*F*). Histology included staining with hematoxylin and eosin, picrosirius red and Masson trichrome, and inflammation scoring. Quantitative collagen percentage calculated using automatic spectral‐segmentation of the staining.

**Statistical Tests:**

Comparing CCl_4_‐treated groups and controls using Welch's t‐test and paired t‐test between different time points. Pearson's correlation used between ROI MEX parameters or high‐*F* fraction, and quantitative histology. *F* or T_1_, and inflammation scores were tested with one‐sided t‐test. *P* < 0.05 was deemed significant.

**Results:**

Rats: *F* values were significantly different after 6 weeks of treatment (0.10 ± 0.02) compared to controls (0.080 ± 0.003). After 8 weeks, *F* significantly increased (0.11 ± 0.02) in treated animals, while controls are not significant (0.0814 ± 0.0008, *P* = 0.079). *F* correlated with quantitative histology (*R* = 0.87), and T_1_ was significantly different between inflammation scores (1: 1332 ± 224 msec, 2: 2007 ± 464 msec).

Mice: *F* was significantly higher (0.062 ± 0.006) in treatment group compared to controls (0.042 ± 0.006). *F* and high‐*F* fraction correlated with quantitative histology (*R* = 0.88; *R* = 0.84). T_1_ was significantly different between inflammation scores (1:1366 ± 99 msec; 2:1648 ± 45 msec).

**Data Conclusion:**

MEX extracted parameters are sensitive to collagen deposition and inflammation and are correlated with histology results of mouse and rat liver fibrosis model.

**Evidence Level:**

1

**Technical Efficacy:**

Stage 3

Liver fibrosis is the result of excessive deposition and accumulation of collagen, proteoglycans, and other macromolecules in the extracellular matrix (ECM).^1^, ^2^, ^3^ Liver fibrosis is a common response to chronic liver injury and may be concomitant with inflammation and steatosis. In the foreseeable future, liver fibrosis is expected to become the leading indication for liver transplantation and liver‐related mortality.^4^ Restoration of normal liver architecture and function is possible with pharmaceuticals, new treatment strategies,^1^, ^3^, ^5^ and lifestyle modifications.^6^, ^7^ Therefore, early diagnosis and periodic staging of liver fibrosis are important for possible blocking or reversing fibrosis.^7^ Currently, the standard clinical method for disease diagnosis and staging is liver biopsy. This invasive procedure includes sampling a small portion of the liver may result with significant pain and bleeding in up to 10.9% of cases.^8^

MRI scans are increasingly used for liver disease diagnosis. Liver disease can be a combination of fibrosis, steatosis, and inflammation with various severities, thus different MRI contrasts and techniques are needed to accurately assess complex conditions. T_2_ and T_1_ relaxation parameters reflect the ability of free water molecules to move inside the ECM, and an elevation in these values is attributed to the greater extent of inflammation.^9^, ^10^ Proton density fat fraction (PDFF) is specific to steatosis estimation and less sensitive to collagen deposition or inflammation.^11^, ^12^ MR elastography (MRE) can detect fibrotic liver, but it is also sensitive to various other causes of stiffening and softening of tissue, such as venous congestion, biliary obstruction, and steatosis.^9^, ^13^ T_1⍴_ and diffusion‐weighted imaging (DWI) are promising approaches and show high sensitivity to motion‐restricted water.^14^ Amide proton transfer (APT) contrast, based on amide bond within the collagen structure, and sodium imaging, which correlates with increased proteoglycans in the ECM, may also hold great potential but are still at early stages of research.^15^ Despite the multiplicity of methods reported, none has become a standard, nor have they replaced the reference standard of liver biopsy.

Magnetization EXchange (MEX) pulse sequence allows the acquisition of a signal generated from macromolecular components of the tissue and from protons adjacent to these components, with quantification analysis of the volumetric fraction of macromolecules.^16^, ^17^ The MEX signal is acquired using selective saturation pulses and spoiling gradients, followed by a recovery period (details in the Materials and Methods section). Typically, MR protons in biological tissues are described by two‐ or three‐pool models, for water content.^18^, ^19^ However, the magnetization detected in the MEX pulse sequence can be divided primarily into contributions from two types of protons: the free water contribution as interacting with the lattice (the fitted T_1_ value), and the contribution of magnetization transferred to the water from non‐aqueous protons (detailed description in the literature^16^, ^17^). MEX pulse sequence was previously used to quantify the myelin fraction in normal brain tissue and demyelination pathology of mice, where the signal collected was attributed to myelin content.^16^, ^17^

The purpose of this study was to test the hypothesis that MEX sequence, and its derived quantitative parameter of macromolecular fraction may be associated with collagen fraction deposition.

## MATERIALS AND METHODS

### Animal Preparation

#### Animal Care and Fibrosis Induction

This research was carried out in accordance with the ethics declaration at the animal care unit (approval # M‐15‐054 and # M‐01‐17‐002).

Sixteen 6‐week‐old male Sprague Dawley rats and 13 C57BL/6 strain male mice were purchased from Envigo RMS (Israel) Ltd. Both mice and rats were included, as the differences in their body weight and dimensions pose a challenge in achieving a selective suppression, warranted by the method.

The rats were randomly divided into four groups: two treatment groups (*n* = 4, *n* = 6) and two control groups (*n* = 3, *n* = 3), corresponding to 6 weeks and 8 weeks of injections. The treated groups and control groups were injected intraperitoneal twice weekly with either 0.3 mL/100 g of carbon tetrachloride (CCl_4_) solution (1:1 ratio CCl_4_:olive oil)^20^ or vehicle, respectively. All rats were scanned after 6 weeks of injections, and then the 6 weeks treatment (*n* = 4) and control (*n* = 3) groups were killed. The two remaining groups (*n* = 6, *n* = 3) were further injected for two more weeks with the same protocol, for a total of 8 weeks, and then scanned and killed for histology.

Mice were divided into two groups, treatment (*n* = 9) and control (*n* = 4) and injected twice weekly for a duration of 8 weeks. Treatment group was administrated with 1 μL/g 1:7 ratio CCl_4_:olive oil solution and controls with vehicle.^20^, ^21^

The protocol of administration provides an established and widely used model of liver fibrosis in rodents by evoking infiltration of inflammatory cells, thus mimicking the changes in chronic viral hepatitis‐associated fibrosis without major fat deposition processes.^20^, ^22^, ^23^

During scans, animals were anesthetized with isoflurane gas 1%–2% in 100% O_2_. Respiration rate was monitored by using a pressure pad positioned under the abdomen and kept at 80–85 bpm in rats and 55–65 bpm in mice. A body temperature of 37 °C was maintained by using a warm water circulation system.

#### Histopathological Procedures

Following the scans, the animals were killed for histological analysis. Livers were excised and fixed with 4% formalin. A portion was then trimmed, embedded in paraffin and sectioned. Rat samples were stained with hematoxylin and eosin (H&E), and picrosirius red (PSR) (*n* = 9) and then scored according the Ishak method^12^ (10 years of experience). Mouse livers (*n* = 12) were stained with H&E and Masson trichrome (MT), and inflammation and morphometrically evaluated by a pathologist (16 years of experience). Pictures were taken using microscope (Olympus BX60) at magnifications of 10× and 40×.

Both PSR and MT allow for collagen visualization as the dyes have good affinity to the collagen fibers by multiple based‐acid interactions.^24^

### Data Acquisition

All scans were performed using a 7 T Bruker BioSpec scanner (Bruker Biospin, Germany) equipped with a commercial 86 mm transmit‐receive volume coil (Bruker), 660 mT/m gradient unit and ParaVision 6.01 imaging software.

#### MEX Sequence

**FIGURE 1:**
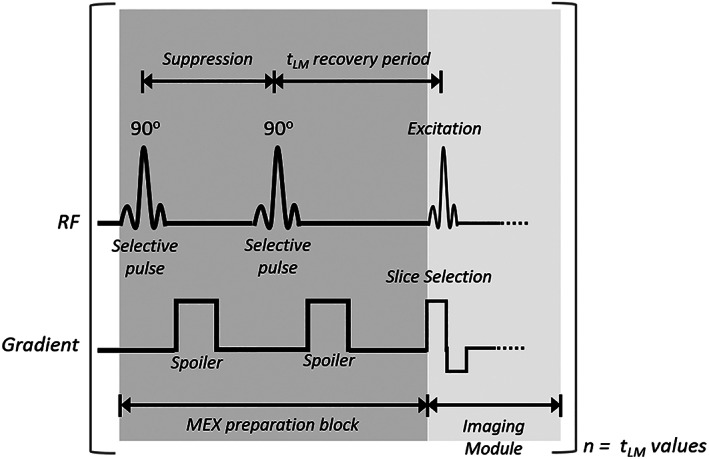
MEX pulse sequence. Two selective pulses are applied at the water resonance, each followed by a spoiler. The delay time, *t*_*LM*_, between the saturation and the imaging module, was varied between repetitions.

Animals were scanned using the proposed MEX sequence as described in Fig. 1. The sequence starts with a suppression block, consisting of two 90° selective pulses (Hermite shaped) and gradient spoilers, followed by a delay time, during which magnetization exchange and recovery occur. When the MEX preparation was complete, a standard imaging module, either SE for rats or GE for mice, was implemented. This technique is different than qMT methods, where the pulses have different offsets and are not selective.^25^, ^26^

To ensure maximal suppression, the pulses power was calibrated by setup scans with fixed short delay time of *t*_*LM*_ = 10 μsec such that minimal signal recovery will occur. The pulses power was varied between scans, and the digitizer filling was monitored to determine the value of highest suppression. The suppression percentage was calculated compared to a similar setup measurement with a long delay time of *t*_*LM*_ = 10 seconds. As the suppression depends on the animal size, the values for the rats were in the range of 97.9%–99.4% and 98.2%–99.6% for the mice (values for each animal are available in Supp. Table S2).

The entire MEX block (suppression and delay time) was performed before each signal acquisition, such that the acquired rows of the k‐space are uniformly weighted by $t_{\mathrm{LM}}$. This experiment was repeated with variations in the delay time ($t_{\mathrm{LM}}$) to acquire the full kinetics of the recovered signal and its dependence on the fraction of the semisolids protons.

#### Rat Scans

The 90° selective Hermite pulses were calibrated to 4.1–5.5 W with fixed duration of 2 msec. Here, a standard spin‐echo imaging module (multi slice multi echo, MSME) was implemented. Nine values of $t_{\mathrm{LM}}$ delays were used: 15, 50, 100, 200, 400, 800, 1600, 2500, and 3000 msec. Five 1‐mm axial slices were acquired with echo time (TE) of 7.2 msec, repetition time (TR) of 1500 msec, 128 × 128 acquisition and reconstruction matrices, and FOV = 57 × 55 mm^2^. The total scan time for each rat was 6 hours 15 minutes to 6 hours 30 minutes (the range depends on the manual calibration time). Additionally, a reference spin‐echo scan (MSME) with similar resolution and delay parameters was acquired.

#### Mouse Scans

The 90° selective Hermite pulses were calibrated to 3.3–3.8 W with fixed duration of 4 msec. Following the recovery period, a gradient‐echo imaging module was employed (fast low‐angle shot, FLASH, with 90^°^ flip angle). Nine values of $t_{\mathrm{LM}}$ delays were used: 15, 25, 50, 75, 100, 200, 600, 1200, and 2500 ms. Two 1‐mm axial slices were acquired with echo time (TE) of 3.2 msec, repetition time (TR) of 3400 msec, 64 × 64 acquisition and reconstruction matrices, and FOV = 40 × 30 mm^2^. Additionally, a reference gradient echo (FLASH) scan with similar resolution and delay parameters was acquired. The total scan time for each mouse was 5 hours 30 minutes to 5 hours 45 minutes (the range depends on the manual calibration time).

### Data Analysis

#### MEX Analysis

Analysis is based on Eq. 1, with the assumption of fast exchange ($\tau_{\mathrm{exc}}\ll T_{1}$) and a flip angle of 90^°^:

(1) $$\frac{M_{\mathrm{zw}}\left(t_{\mathrm{LM}}\right)}{M_{\mathrm{ZW}}^{\mathrm{eq}}}=F\left(1-\exp\left(\frac{t_{\mathrm{LM}}}{\tau_{\mathrm{exc}}}\right)\right)+\left(1-F\right)\left(1-\exp\left(\frac{\;t_{\mathrm{LM}}}{T_{1}}\right)\right)$$

$M_{\mathrm{ZW}}^{\mathrm{eq}}$ is the water equilibrium z‐magnetization, *F* is the fraction of protons in semisolids, $\tau_{\mathrm{exc}}$ is the exchange time, and T_1_ is the longitudinal relaxation time. For very short delays ($t_{\mathrm{LM}}\ll T_{1}$), the first term of Eq. 1 dominates, that is, the image intensity reflects the content of the immobile species.^16^, ^17^

The acquired images were normalized by the longest $t_{\mathrm{LM}}$ scan and fitted to Eq. 2 using nonlinear least squares Trust‐Region algorithm in MATLAB R2021b (MathWorks, MA).

(2) $$\frac{S\left(t_{\mathrm{LM}}\right)}{S\left(t_{\mathrm{LM}}=t_{\mathrm{LM}}^{\max}\right)}=\frac{F\left(1-\exp\left(\frac{t_{\mathrm{LM}}}{t_{\mathrm{exc}}}\right)\right)+\left(1-F\right)\left(1-\exp\left(\frac{t_{\mathrm{LM}}}{T_{1}}\right)\right)}{F\left(1-\exp\left(\frac{t_{\mathrm{LM}}^{\max}}{t_{\mathrm{exc}}}\right)\right)+\left(1-F\right)\left(1-\exp\left(\frac{t_{\mathrm{LM}}^{\max}}{T_{1}}\right)\right)}$$

The quality of fit was assessed based on the *R* ^2^ value of the fit, and the extracted parameters, *F* and T_1_, were included only for voxels meeting the condition of *R* ^2^ > 0.95. Histograms of *F* maps were generated, and the percentage of voxels with *F* values in the upper end of the range (*F*>0.1) was calculated.

**FIGURE 2:**
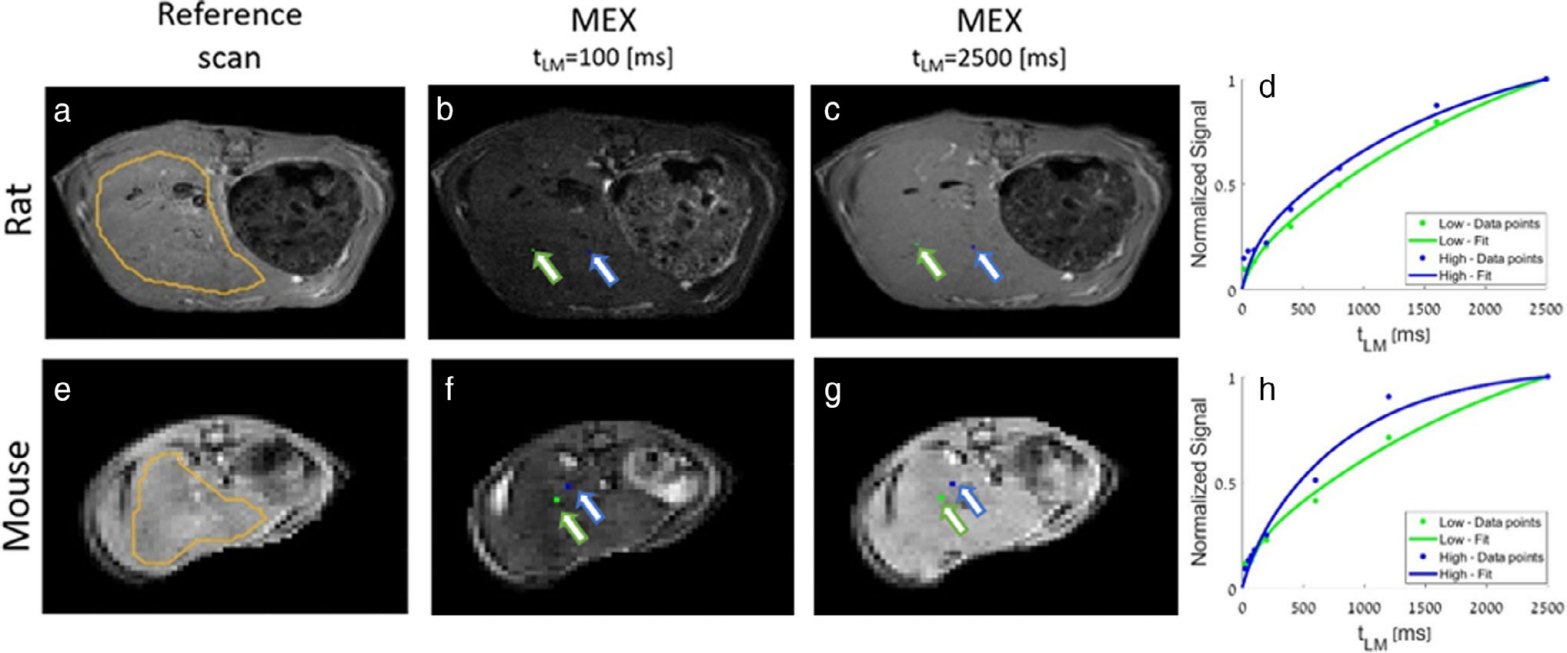
Reference anatomical MRI scans and MEX‐weighted scans in an example rat (top row) and mouse (bottom row). (a) SE and (e) GE reference scans, with liver ROI manually marked for rat and mouse, respectively. (b) and (f) are MEX scans at short delay time of *t*_*LM*_ = 100 msec. (c) and (g) are MEX scans at the longest delay time, *t*_*LM*_ = 2500 msec. Plots (d) and (h) are the normalized signal of the specific pixels marked in (b), (c), (f), (g), as a function of *t*_*LM*_. The green pixels were fitted with relatively low macromolecular fraction (*F* = 0.004 in (d) and *F* = 0.03 in (h)) and blue with relatively high macromolecular fraction (*F* = 0.22 in (d) and *F* = 0.14 in (h)).

Additionally, the liver area was manually segmented (by E. S., 10 years of experience) based on the reference scans (see, eg Fig. 2a,e), and only voxels within this regions of interest (ROI) were included in any further calculations and statistical analysis. Voxels with high liquid content and very low macromolecular fraction (such as blood vessels) that do not meet the *R* ^2^ quality value condition were thus automatically excluded from the ROI by the algorithm. The signal in the entire liver volume was summed and fitted to Eq. 2 again to obtain an *R* ^2^ > 0.999 for the fit and used in the group analysis.

#### Histology Image Quantification

For a quantitative evaluation, the histology images were segmented automatically, using the Color Thresholder application in MATLAB. Images were transformed from RGB to YCbCr color space, and based on the color spectra of each stain, specific thresholds to each channel were defined. For PSR, the values were: [Y Cb Cr] = [0:184 121:144 136:255] and for MT, values were: [Y Cb Cr] = [67:255 129:255 119:159]. Following the segmentation, a mask was generated and the relative fraction of the collagen was calculated for each image.

#### Statistical Analysis

As the data are normally distributed (details in Supp. Table S1), an unpaired Student's *t*‐test with unequal variance (Welch's *t*‐test) was used to compare independent variables of each group (fibrosis and control), and paired *t*‐test to assess the progression between the two time points. When *F* and T_1_ were compared to inflammation scoring, one‐sided *t*‐test was used. Correlations between MEX parameters and histopathology were evaluated using Pearson's correlation. Significance was determined by the upper and lower prediction interval of 0.95, and *P*‐values below 0.05 were considered significant. Fitted parameters of the algorithm appear as mean ± CI (95% confidence interval of the fit), otherwise, values appear as mean ± SD.

## RESULTS

### Histopathology Images and Segmentation

**FIGURE 3:**
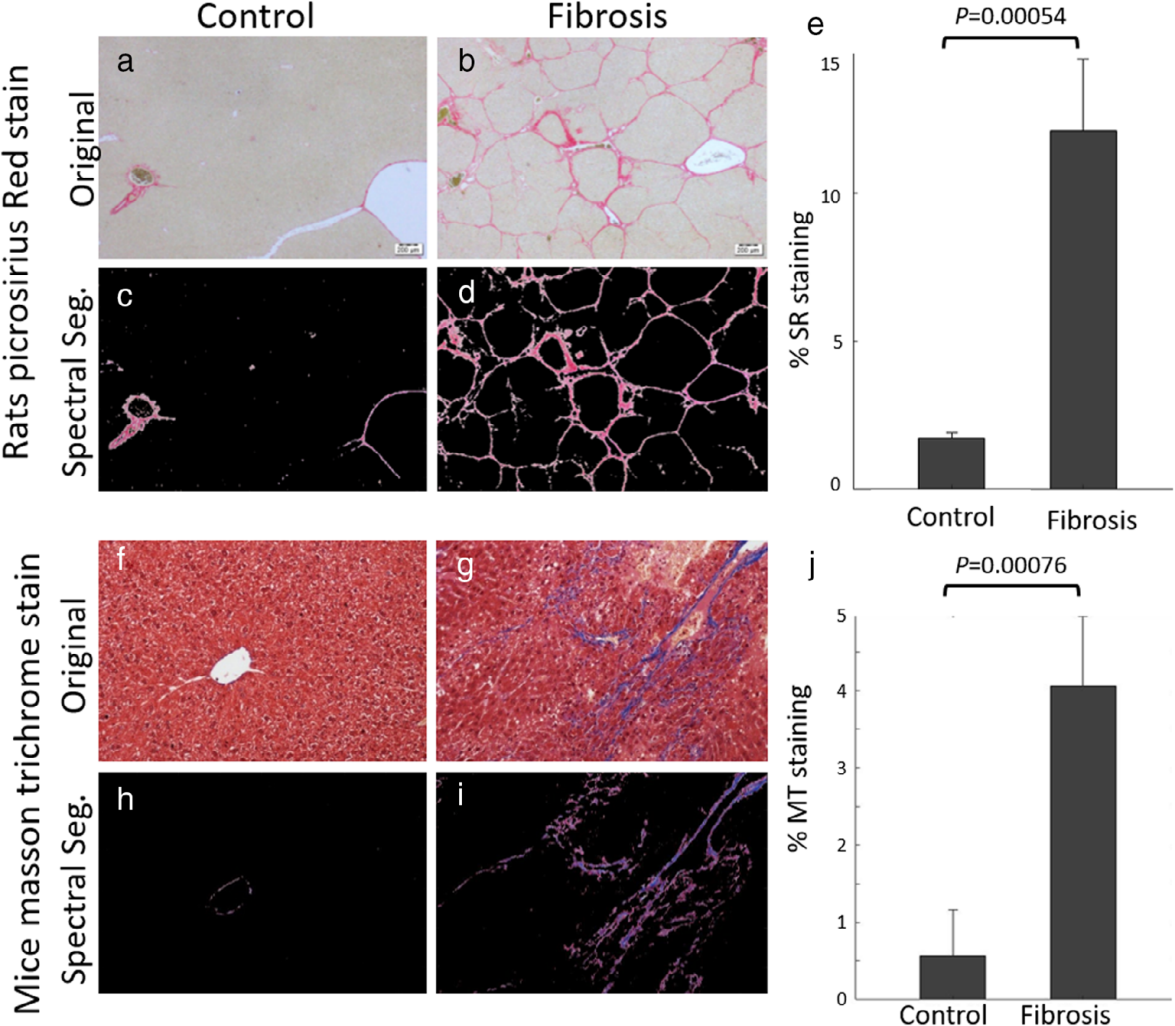
Histology images of liver samples stained with H&E and PSR, for control (a) and CCl_4_‐treated (b) rats; (c) and (d) are the spectral segmentation of the images, showing the red color of the collagen deposition in control and treated, respectively; (e) shows the average PSR percentage of stained pixels between control and treatment groups. The histology images for mouse liver samples were stained with H&E and MT. Panels (f) and (g) are for control and CCl_4_ treated and (h) and (i) are the spectral segmentation, respectively; (j) is the MT staining percentage between the groups.

The Ishak staging based on the PSR staining and of rats treated with CCl_4_ was 4.0 ± 0.9, which is characterized as “fibrous expansion of portal areas with marked bridging (portal‐portal and portal‐central)”,^12^ and controls were scored 0 (no fibrosis). Inflammation scoring for the treated animals was either 1 (very‐mild, cells<10) or 2 (mild, 10 cells<20), and 0 (absent) for controls. After automated spectral segmentation, the percentage of PSR staining (fibrotic tissue) was significantly different between CCl_4_‐treated rats and controls, 11.87% ± 0.03% and 1.972% ± 0.003%, respectively (see Fig. 3a–e).

Mouse liver samples with MT staining had morphometric score of 8.6 ± 5.7 for the fibrosis group and 1.2 ± 1.6 for controls. Inflammation scoring for the treated animals was either 1 or 2, and 0 for controls. Quantitative evaluation of the automated spectral segmentation yielded 4.08% ± 0.02 fibrotic tissue for CCl_4_‐treated mice, and 0.573% ± 0.006 for controls, see Fig. 3f–j.

### MEX Imaging

Example MEX weighted images at 2 *t*_*LM*_ times are depicted in Fig. 2b,c,f,g. Two specific voxels exhibiting higher (*F* = 0.22 and F = 0.19) and lower (*F* = 0.004 and *F* = 0.02) macromolecular fractions, for rat and mouse, respectively, were chosen. Their normalized signal as a function of delay time was plotted in Fig. 2d,h, to show the full dynamic and its difference.

#### Rats Imaging

**FIGURE 4:**
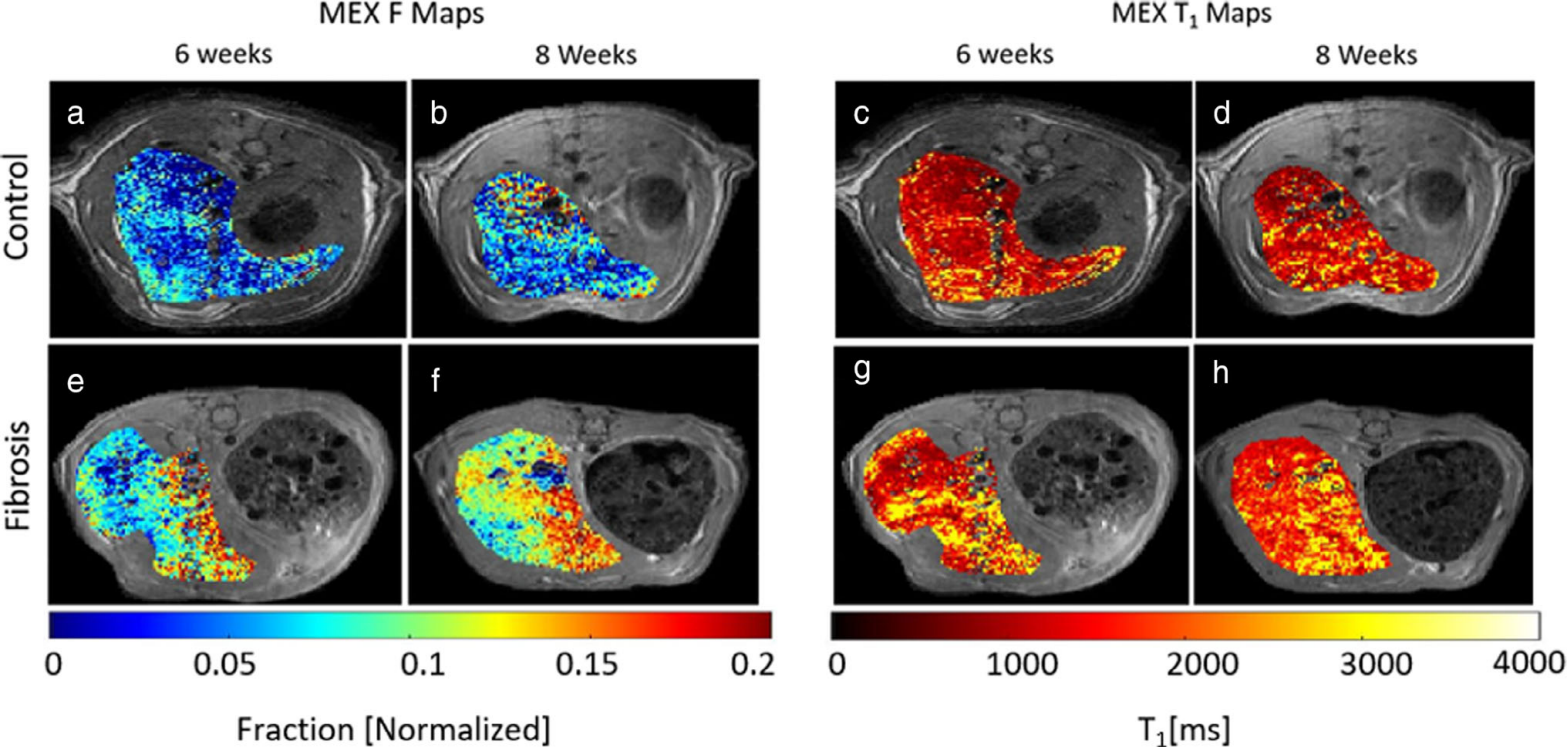
Example MEX fitted maps for control (top) and fibrosis (bottom) rats, each voxel with *R* ^2^ > 0.95. Fitted *F* maps (a,b) and T_1_ maps (c,d) for a control rat at 6 and 8 weeks, respectively. Fitted *F* maps (e,f) and T1 maps (g,h) for a fibrosis rat after 6 and 8 weeks of treatment, respectively.

The data collected using MEX were analyzed through the pipeline described, and full maps of fitted parameters, *F* and T_1_, are shown in Fig. 4, for representative animals.

Figure 4a,b is the F maps of a control rat after 6 and 8 weeks of injections with whole ROI fitted values of 0.083 ± 0.005 and 0.084 ± 0.005, respectively (mean ± CI). Figure 4e,f is the *F* maps for a CCl_4_ treated rat, with fitted values after 6 weeks of 0.125 ± 0.006, and after 8 weeks of 0.131 ± 0.008 (mean ± CI). The fitted T_1_ maps appear in Fig. 4c,d for a control rat and Fig. 4g,h for a treated rat, after 6 or 8 weeks of injection, with whole ROI fitted values of 1474 ± 63 (msec), 1591 ± 38 (msec), 1844 ± 12 (msec) and 1833 ± 14 (msec) (mean ± CI), respectively.

**FIGURE 5:**
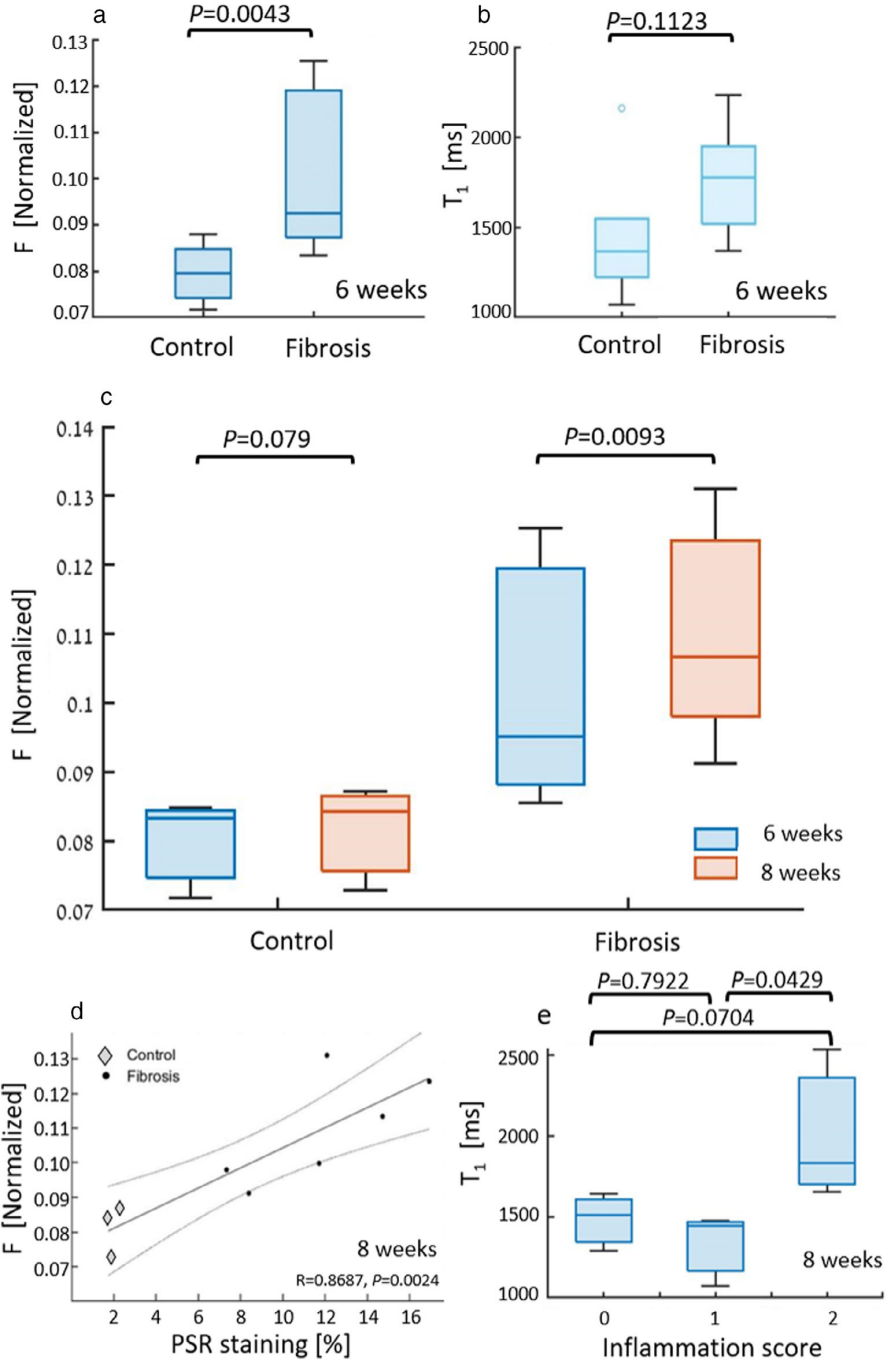
*F* and T_1_ parameters for all CCl_4_‐treated and control rats. (a) Mean *F* values in the fibrosis group (*n* = 10) are higher by 24.7% (*P* < 0.005) than control (*n* = 6); (b) mean T_1_ values are higher in the fibrosis group by 22.2% (*P* = 0.1123). (c) Kinetics of the derived F parameter for two scan time points. *F* values compared between the two subgroups of control (*n* = 3) and fibrosis (*n* = 6) that were scanned at 6 weeks (blue) and 8 weeks (red). The fibrosis group with an average increase of 7.9% (*P* < 0.01), and the control increased by 1.8% (*P* = 0.079); (d) is a correlation chart between the histology staining percentage based on the spectral segmentation to *F* (*R* = 0.8687; *P* < 0.005). Circles are control and rectangles are fibrosis mice. Dotted lines are the prediction intervals, refer to upper and lower 95% prediction bounds; (e) is a comparison between T_1_ and the inflammations, with values of: 0: 1481 ± 177 msec; 1: 1332 ± 224 msec; 2: 2007 ± 464 msec.

Figure 5a,b displays the group analysis for all groups of both *F* and T_1_ ROI fitting, at the 6 weeks' time‐point. Mean *F* value is significantly increased by 24.7% in the treated group compared to control (CCl_4_: 0.099 ± 0.016; control: 0.080 ± 0.003), while T_1_ is higher by 22.2% but not significantly (*P* = 0.11). Figure 5c is a comparison of the fitted *F* values of the 8 weeks injection groups, between the first and second scans. The values of fibrotic rats after 8 weeks of injections is significantly higher by 7.9% compared to the 6 weeks scan time (CCl_4_: 0.11 ± 0.02), while control rats show 1.8% nonsignificant increase (0.0814 ± 0.0008, *P* = 0.08).

Figure 5d,e depicts the comparison of the MEX fitted parameters after 8 weeks of injections to the histology analysis. In Fig. 5d,f, values are correlated with the collagen percentage based on the automated spectral segmentation of the PSR staining (*R* = 0.87). Similar correlation of T_1_ and collagen percentage from PSR staining (*R* = 0.50, *P* = 0.17) is available in Supp. Fig. S1a. In Fig. 5e, a comparison between the fitted T_1_ and the inflammations score is plotted. While the T_1_ difference between scores 0 and 1 is not significant (*P* = 0.79), the T_1_ values increase by 26.2% from no to mild inflammation (not significant*, P* = 0.07), and from very mild to mild increased significantly by 33.7% (0: 1481 ± 177 msec; 1: 1332 ± 224 msec; 2: 2007 ± 464 msec). Complementary comparisons between *F* values and inflammation appear in Supp. Fig. S1b, where the differences are nonsignificant within the fibrosis group (scores of 1 vs. 2, *P* = 0.13), only significant between control and fibrosis (0 vs. 1 and 2). The analysis normalized histograms of *F* maps and the percentage of pixels with 0.1 < *F* with correlation to quantitative collagen percentage from PSR staining (*R* = 0.84) is available in Supp. Fig. S2.

#### Mouse Imaging

**FIGURE 6:**
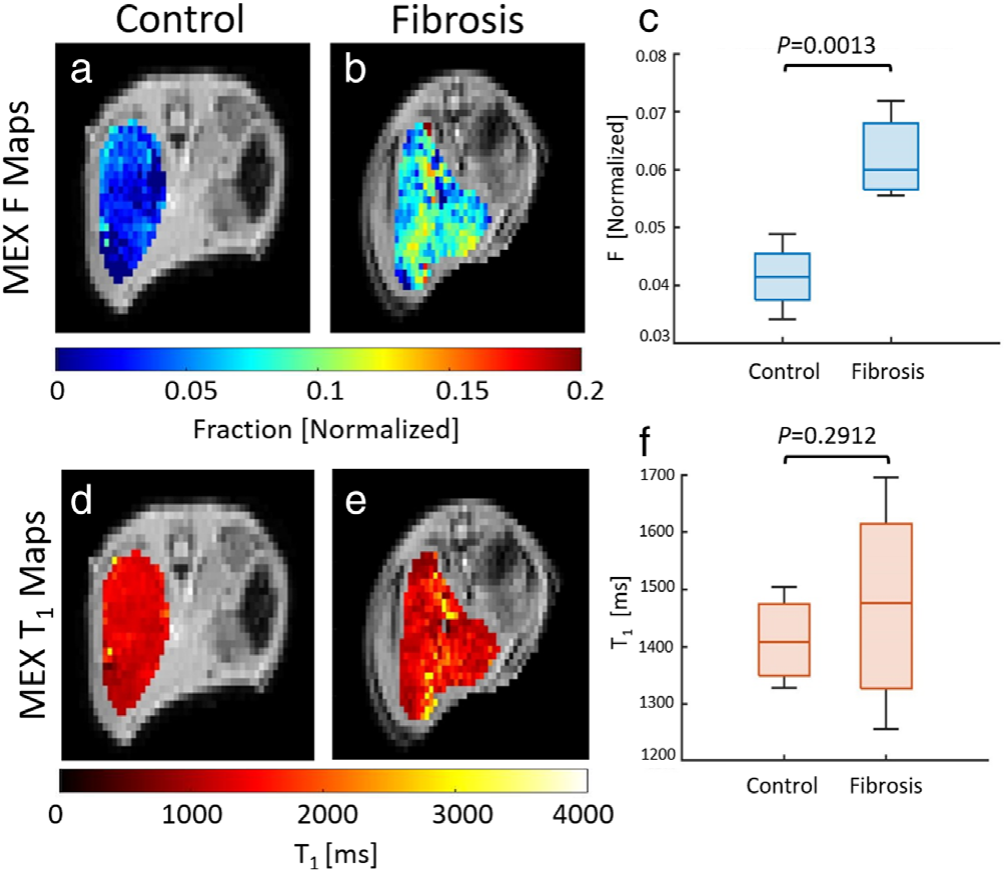
Example MEX *F *maps (top) and T_1_ maps (bottom) of control (a,d) and CCl_4_ treated mice (b,e), each voxel with *R* ^2^ > 0.95; (c) and (f) are the group analysis for both fitted parameters. *F* value is increased by 49.7% (*P* < 0.005) and T_1_ is increased by 5.2% (*P* = 0.2912).

The MEX fitted parameters *F* and T_1_ maps are shown in Fig. 6a,b,d,e, for representative animals. The whole ROI *F* value of the control mouse is 0.034 ± 0.009 and 0.072 ± 0.003 for the CCl_4_‐treated mouse (mean ± CI). The whole ROI T_1_ values are 1335 ± 23 msec and 1427 ± 60 msec for the control and fibrosis mice, respectively (mean ± CI). The group analysis for both *F* and T_1_ appears in Fig. 6c,f. The *F* value increased significantly by 49.7% between treatment and control groups (CCl_4_: 0.062 ± 0.006, control: 0.042 ± 0.006), while the T_1_ value showed an insignificant rise of 5.2% (CCl_4_: 1485 ± 160 msec, control: 1436 ± 54, *P* = 0.29).

**FIGURE 7:**
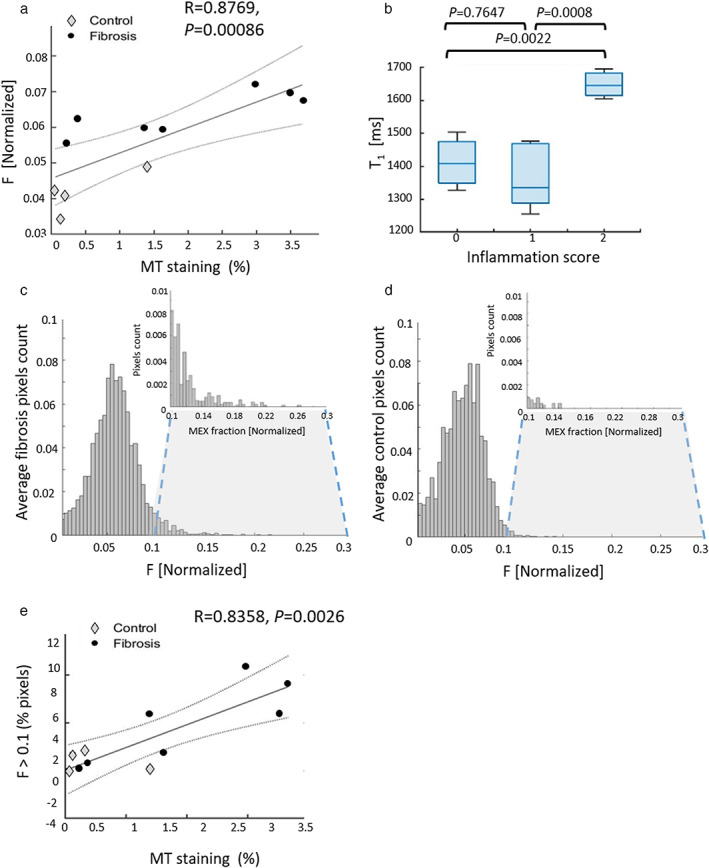
Correlation chart between the histology staining percentage based on the spectral segmentation to *F* (*R* = 0.8769; *P* < 0.001) (a) and T_1_ comparison to inflammation with values 0: 1436 ± 54 msec, 1: 1366 ± 99 msec, 2: 1648 ± 45 msec (b). Average histograms of *F* maps for fibrosis (c) and control (d) normalized by total number of pixels. The insert is a zoomed‐in display of the pixels in the range [0.1–0.3]. Based on the histograms of each animal, the percentage of pixels with *F* values in the upper range [0.1–0.3] was calculated and correlated with the histology staining percentage shown in (e) (*R* = 0.8384, *P* < 0.005). Circles are control and rectangles are fibrosis mice. Dotted lines are the prediction intervals, refer to upper and lower 95% prediction bounds.

In Fig. 7a,b, comparisons of the MEX fitted parameters to the histology analysis are depicted. *F* values showed a good correlation to the percentage of collagen based on the histology spectral analysis (*R* = 0.88). The T_1_ correlation to collagen percentage appears in Supp. Fig. S1c (*R* = 0.07; *P* = 0.86). The comparison of T_1_ to the inflammation score shows, similar to that of rats, no significant change of values between no inflammation to very mild inflammation (*P* = 0.77), while significantly increasing by 17.1% within the fibrosis group between very mild and mild inflammation (0: 1436 ± 54 msec, 1: 1366 ± 99 msec, 2: 1648 ± 45 msec). *F* comparison to inflammation was not significant between inflammation scores (1 vs. 2, *P* = 0.43), but rather only significant between control and fibrosis (appears in Supp. Fig. S1d). Additionally, based on normalized histograms of *F* maps (average histograms in Fig. 7c,d), the percentage of pixels with 0.1 < *F* was correlated with the MT staining percentage (*R* = 0.84).

All the values for rats and mice are available in Supp. Table S2.

## DISCUSSION

The MEX MRI sequence acquires signal originating from macromolecules in the tissue, by applying selective saturation pulses and spoiling gradients, and monitoring the signal recovery. This approach assumes magnetization exchange between macromolecular content as well as spin–lattice interaction.^16^ Therefore, when T_1_ is measured by either saturation or inversion recovery sequences, where the data are analyzed with the assumption of spin–lattice interaction alone, the extracted value actually captures more complex interactions.^16^ Thus, the T_1_ values extracted from the MEX are not comparable to values extracted from mono‐exponential fits, as it is sensitive mainly to the water interactions. Other quantitative magnetization transfer‐based methods^27^, ^28^, ^29^ also capture the complex mechanisms, but they use seven or five parameters to describe the spin kinetics. Moreover, in order to achieve a more robust fitting, Monte Carlo simulations are preformed to ensure convergence. In the MEX analysis, there is no need for prior estimations due to the low number of fitted parameters. It should be noted that in these clinical studies, the method was combined with fast imaging modules,^27^, ^28^, ^29^ which are still being developed for MEX.

### Rats

The CCl_4_ administration protocol for the rat model yielded a significant deposition of collagen as captured by the Ishak's scoring and quantitative spectral segmentation of the PSR staining images, and very mild to mild inflammation. Steatosis (fat deposition) was not evaluated, but is known to be insignificant in this model after 6 or 8 weeks.^20^, ^22^, ^23^, ^30^ *F* values for CCl_4_‐treated groups were significantly higher at the 6 weeks' time point compared to the controls. Values continued to increase, significantly, at the 8 weeks' time point, while the control groups did not show significant changes. Moreover, a variation in response to the CCl_4_ injections was measured in the treated groups in both time points, with high variance of the values, which was not evident for controls. Although, only the 8 weeks' time point groups were evaluated with histology, the *F* values correlated with the quantitative percentage of the collagen, based on PSR staining. In this correlation, the linear fit does not cross the origin, presumably due to other macromolecular components of the tissue. The value of the *F* is, thus, sensitive to changes in the relative collagen deposition.

The T_1_ values did not change significantly across groups and also did not correlate with the percentage of collagen extracted from the histology‐stained images. Although T_1_, when measured by saturation or inversion recovery, appears to be a relatively good predictive parameter for fibrosis and inflammation,^15^, ^31^ MEX fitted T_1_ parameter, as detailed above, is not comparable. It is mainly sensitive to the water content of the tissue, as evident by the significant changes measured between very mild and mild inflammation scores. It is worth noting that between the controls (no inflammation) and the very mild inflammation the changes were not significant. This might be due to competing interaction of the fibrosis and inflammation, as the two factors do not necessarily increase together in each rat, and the small sample group.

Previous works, by Hu et al^32^ and Zhang et al,^14^ with same CCl_4_ administration protocol in Sprague Dawley rat model, captured significant changes in T_1⍴_, T_2_ and ADC values after 5–10 weeks. In contrast, Xie et al,^33^ who scanned six time points with similar protocol, were unable to detect significant difference in T_1⍴_ values between their second and third time points (corresponding to 6 and 8 weeks in this study). The results presented for MEX rat model match the same time‐frame for detection (5–10 weeks) as presented by the above studies,^14^, ^32^ indicating that the sensitivity to fibrosis and inflammation is similar. MRE is another MR contrast that allows for liver stiffness measurement. Importantly, it is very sensitive to acute inflammation and necrosis, even when no fibrosis is detected,^9^ as opposed to the separation of fibrosis and inflammation that are possible by MEX.

### Mice

MEX scan of mice used GE imaging modules, opposed to SE in rats. The two different imaging sequences differ in the lower T_2_ weighting in GE (shorter possible TE), as well as the correction for B_0_ inhomogeneity. Nonetheless, both animal models showed good correlations of the extracted values to quantitatively histology analysis, demonstrating the versatility of the method, and its low dependence on the imaging module. Additionally, the tissue assessment using histopathology demonstrated lower development of fibrosis in mice relative to that appears in rats, while keeping the same inflammation score. Both findings were captured by the two MEX parameters *F* and T_1_.

Mouse imaging suffered from lower resolution; nonetheless, significant differences in the *F* values were apparent between the treated and the control groups. Furthermore, evaluation of all mice with histopathology was possible, and correlation of *F* values with collagen percentage from the MT staining was good. Pixels exhibiting high‐*F* values (based on the histograms) are assumed to have higher fraction of collagen, thus their significant correlation to the histology spectral segmentation further supports the *F* values sensitivity to fibrosis.

Similar to the results in rats, the T_1_ values changes significantly between very mild and mild inflammation scores, strengthening the different mechanisms captured, as opposed to mono‐exponentially fitted T_1_. The divided attribution of the MEX signal between the extracted *F* and T_1_ is also validated by the lack of significant change of *F* values within treated group, with respect to inflammation.

Mouse CCl_4_ administration protocols are more diverse,^15^, ^22^, ^34^ thus require caution when compared. Müller et al^34^ were able to detect significant changes in T_1_ and T_2_* after 3 weeks, but with 5.5 times higher accumulated dose than that used here (comparable to 16.5 weeks in this study). Chow et al^22^ showed significant increase in T_1_ and T_2_ after 2 weeks, though administrated 8× times the CCl_4_ dose in this study (corresponding to 16 weeks). Also, Lindquist et al^15^ found significant T_1_ elevation only after 12 weeks with 1.25 times the conditions presented here (corresponding to 15 weeks). In their work,^15^ values of T_1⍴_ and T_2_ did change significantly at both scan time points (12 and 16 weeks). All three of these studies,^15^, ^22^, ^34^ when normalized to doses and duration, found the significant T_1_ value after 15–16 weeks. In this MEX study, significant *F* values were found to increase after 8 weeks of treatment. This implies that the MEX may be sensitive to the early stages of fibrosis development, relative to other methods. This makes it a potentially good biomarker for fibrosis. It should be noted that although the accumulated doses were normalized, tissue response to the protocol (such as inflammation) might be different when each injection contains higher CCl_4_ concentration (higher rate of administration).

### Limitations

The MEX experiment calls for sequential scans with different delay times ($t_{\mathrm{LM}}$) of up to 3000 msec, an added time to the TR. The overall scan for a single animal is therefore relatively long, forcing a lower resolution and liver coverage. This poses an obstacle when clinical studies are considered. To that end, combining with fast imaging sequences is being developed (e.g. echo planar imaging). This was an animal study, rather than a human study, thus limited in the inflicted liver disease characteristics, mainly due to the lack of steatosis. It is impossible to determine, in more complex scenarios, whether the extracted *F* value will allow the distinction between multiple sources of macromolecular changes (protein and lipids). Note that in previous studies, it was suggested that those semi‐solids microstructures should be singly oriented in order to transfer magnetization acquired by MEX.^16^, ^17^

The selective pulses used during the preparation module of the scan were calibrated before each animal was scanned to achieve maximal water suppression, as the method requires. This calibration cannot always yield exact results; consequently, there were some changes to the signal recovery curve. Although these changes can result in up to 2% deviation of the extracted values, they could have affected the correlation between the MEX results and the histology analysis, as well as other comparisons.

Another limitation is repeatability: we did not evaluate scan–rescan measurements in each specific time point in the mouse and rat studies. Nonetheless, we did evaluate the rat model at two time points, and relied on previous publications involving MEX,^16^, ^17^ in addition to the histology validation. As liver is a very large organ, and since the histology performed did not cover the entire scanned volume, the comparison between the two methods is limited. As evident by the MEX maps, there is a variability in fibrosis severity in different areas; therefore, the specific location of the histology slide limits an accurate correlation. Finally, our histology analysis for the rat model was not complete, thus histology validation was possible only at the 8 weeks' time point.

### Conclusion

The fitted *F* value indicated the relative percentage of collagen in the liver. It might be a good biomarker for fibrosis, as it has low sensitivity to other tissue changes, such as inflammation, which is captured by the extracted MEX T_1_.

This study implemented the MEX sequence and analysis on the liver tissue of two animal models. The method was previously implemented ex vivo and in vivo only on brain tissues.^16^, ^17^ Following the promising results in this study and the previous ones, the MEX sequence and its derived parameters were proven to be sensitive to various tissues and macromolecules, making it a possible quantitative imaging method to many other pathologies.

## Supporting information


Appendix S1 Supporting Information
