# Supplementary material for: Quantitative Magnetization EXchange MRI Measurement of Liver Fibrosis Model in Rodents
Source: J Magn Reson Imaging. 2022 May 6;57(1):285–95. doi: 10.1002/jmri.28228 (PMC10084184; doi:10.1002/jmri.28228)
Supplement: Supplementary file 1 — Appendix S1 Supporting Information [file JMRI-57-285-s001.docx]

**Supporting Information**

***Statistical Analysis***

The statistical significance in this study was determined using student's t-test as the data is normally distributed. Table S1shows the D'Agostino & Pearson normality test results.

**Table S1 – D'Agostino & Pearson normality test for cuprizone and control groups**

| D'Agostino & Pearson normality test | Fibrosis (Rats) | Control (Rats) | Fibrosis (Mice) | Control (Mice) |
| --- | --- | --- | --- | --- |
| K2 | 0.6082 | 1.070 | 0.5924 | 0.9836 |
| P value | 0.7856 | 0.5982 | 0.6993 | 0.5749 |
| Passed normality test (α=0.05)? | YES | YES | YES | YES |
| P value summary | ns | ns | ns | ns |

***Data Results***

In Sup. Table S2 the fitted F value and T_1_ for both mice and rats are displayed, alongside the collagen fraction based on spectral segmentation, inflammation score and the water saturation percentage, for all animals.

**Table S2 – F values, T_1_ values and histology analysis of All Animals**

| Animal # | Supp. | F value | | | | Histogram | | | T_1_ [ms] | | | | | | | Histology | | |
| --- | --- | --- | --- | --- | --- | --- | --- | --- | --- | --- | --- | --- | --- | --- | --- | --- | --- | --- |
|  |  | 6 weeks | | 8 weeks | | | pixels with  0.1<F | 6 weeks | | | 8 weeks | | | Collagen  Spect. seg.  [%] | | | Inflam. score | |
|  |  | Mean | ±CI | Mean | ±CI | |  | Mean | | ±CI | | Mean | ±CI | |  | | |  |
| RatFib01 | 98.5% | 0.0873 | 0.0029 | - | - | | 45.55% | 1439.95 | | 79.83 | | - | - | | - | | | - |
| RatFib02 | 97.9% | 0.1190 | 0.0059 | - | - | | 59.89% | 1951.57 | | 26.03 | | - | - | | - | | | - |
| RatFib03 | 99.4% | 0.0950 | 0.0032 | - | - | | 38.28% | 1713.47 | | 44.17 | | - | - | | - | | | - |
| RatFib04 | 98.0% | 0.0835 | 0.0042 | - | - | | 21.31% | 1369.18 | | 31.28 | | - | - | | - | | | - |
| RatFib05 | 99.1% | 0.1253 | 0.0056 | 0.1310 | 0.0075 | | 14.00% | 1844.68 | | 12.28 | | 1833.54 | 14.09 | | 12.09% | | | 2 |
| RatFib06 | 98.5% | 0.1194 | 0.0047 | 0.1235 | 0.0064 | | 19.50% | 1874.98 | | 10.59 | | 2534.88 | 55.25 | | 16.93% | | | 2 |
| RatFib07 | 99.1% | 0.0999 | 0.0055 | 0.1134 | 0.0057 | | 20.13% | 1696.40 | | 84.94 | | 1476.35 | 68.86 | | 14.72% | | | 1 |
| RatFib08 | 98.3% | 0.0902 | 0.0084 | 0.0980 | 0.0032 | | 32.43% | 1519.34 | | 82.86 | | 1655.52 | 98.30 | | 7.34% | | | 2 |
| RatFib09 | 98.5% | 0.0855 | 0.0031 | 0.0999 | 0.0011 | | 28.88% | 2234.55 | | 26.98 | | 1073.91 | 45.20 | | 11.72% | | | 1 |
| RatFib10 | 99.0% | 0.0882 | 0.0035 | 0.0912 | 0.0081 | | 26.46% | 2164.26 | | 11.29 | | 1445.76 | 77.26 | | 8.39% | | | 1 |
| RatCont01 | 98.1% | 0.0742 | 0.0026 | - | - | | 29.36% | 2161.86 | | 23.49 | | - | - | | - | | | - |
| RatCont02 | 99.3% | 0.0759 | 0.0025 | - | - | | 37.90% | 1258.65 | | 17.89 | | - | - | | - | | | - |
| RatCont03 | 98.7% | 0.0880 | 0.0028 | - | - | | 18.94% | 1550.68 | | 28.62 | | - | - | | - | | | - |
| RatCont04 | 99.1% | 0.0833 | 0.0052 | 0.0843 | 0.0046 | | 12.83% | 1474.16 | | 63.17 | | 1591.56 | 38.80 | | 1.73% | | | 0 |
| RatCont05 | 99.0% | 0.0848 | 0.0039 | 0.0872 | 0.0033 | | 12.93% | 1224.21 | | 29.22 | | 1511.16 | 42.39 | | 2.30% | | | 0 |
| RatCont06 | 98.2% | 0.0717 | 0.0096 | 0.0728 | 0.0032 | | 20.41% | 1073.09 | | 41.20 | | 1641.93 | 12.36 | | 1.89% | | | 0 |
| MiceFib01 | 99.3% | - | - | 0.0719 | 0.0031 | | 11% | - | | - | | 1335.96 | 23.89 | | 2.97% | | | 1 |
| MiceFib02 | 98.7% | - | - | 0.0569 | 0.0014 | | - | - | | - | | 1476.60 | 13.95 | | 7.58% | | | 1 |
| MiceFib03 | 98.4% | - | - | 0.0694 | 0.0024 | | 5.96% | - | | - | | 1465.83 | 33.01 | | 4.52% | | | 1 |
| MiceFib04 | 98.9% | - | - | 0.0627 | 0.0025 | | 7.5% | - | | - | | 1644.61 | 23.77 | | 3.69% | | | 2 |
| MiceFib05 | 99.3% | - | - | 0.0597 | 0.0033 | | 5.98% | - | | - | | 1695.35 | 23.35 | | 3.38% | | | 2 |
| MiceFib06 | 98.2% | - | - | 0.0535 | 0.0026 | | 9.09% | - | | - | | 1604.90 | 17.22 | | 5.67% | | | 2 |
| MiceFib07 | 99.6% | - | - | 0.0600 | 0.0026 | | 1.83% | - | | - | | 1299.86 | 37.18 | | 2.62% | | | 1 |
| MiceFib08 | 98.7% | - | - | 0.0558 | 0.0025 | | 2.31% | - | | - | | 1590.66 | 29.32 | | - | | | - |
| MiceFib09 | 98.3% | - | - | 0.0675 | 0.0021 | | 3.1% | - | | - | | 1255.55 | 24.86 | | 2.24% | | | 1 |
| MiceCont01 | 99.1% | - | - | 0.0409 | 0.0009 | | 0.23% | - | | - | | 1503.92 | 11.66 | | 0.18% | | | 0 |
| MiceCont02 | 99.3% | - | - | 0.0489 | 0.0025 | | 0.24% | - | | - | | 1445.43 | 22.23 | | 0.12% | | | 0 |
| MiceCont03 | 98.2% | - | - | 0.0342 | 0.0086 | | 1.67% | - | | - | | 1427.39 | 60.69 | | 1.40% | | | 0 |
| MiceCont04 | 99.4% | - | - | 0.0421 | 0.0031 | | 0.1% | - | | - | | 1371.20 | 26.50 | | 0.59% | | | 0 |

Abbreviations: Cont. indicates the control group; Fib, the CCl_4_ treated group; Supp. is the percentage of water suppression after the MEX preparation, of the maximal digitizer filling; CI is the 95% confidence interval of the fit; Spect. seg. is the automatic spectral segmentation of collagen and Inflam. is the inflammation score given by the pathologist.

*F and T_1_ Parameters compared with histology*

None significant correlations between T_1_ extracted value and collagen percentage based on automated spectral segmentation of the histology staining appears in Fig. S1a for rats (R=0.4996, *P*=0.1709) and Fig. S1c for mice (R=-0.0663, *P*=0.8557). The comparison between F values and inflammatory score for rats and mice are depicted in Fig. S1b,d, respectively. In both rats and mice the significant differences (*P*<0.05, *P*<0.005, respectively) are between control (0 score) and fibrosis (score of 1 or 2), and not significant (*P*=0.1304, *P=*0.4341) within the treated groups (1 vs. 2) themselves. Rats F values are for different inflammation scores are: absent: 0.0814±0.0076, very mild: 0.1015±0.0111, mild: 0.1175±0.0173; and for mice: absent: 0.0415±0.0060, very mild: 0.0627±0.0075 [ms], mild: 0.0634±0.0038 [ms].


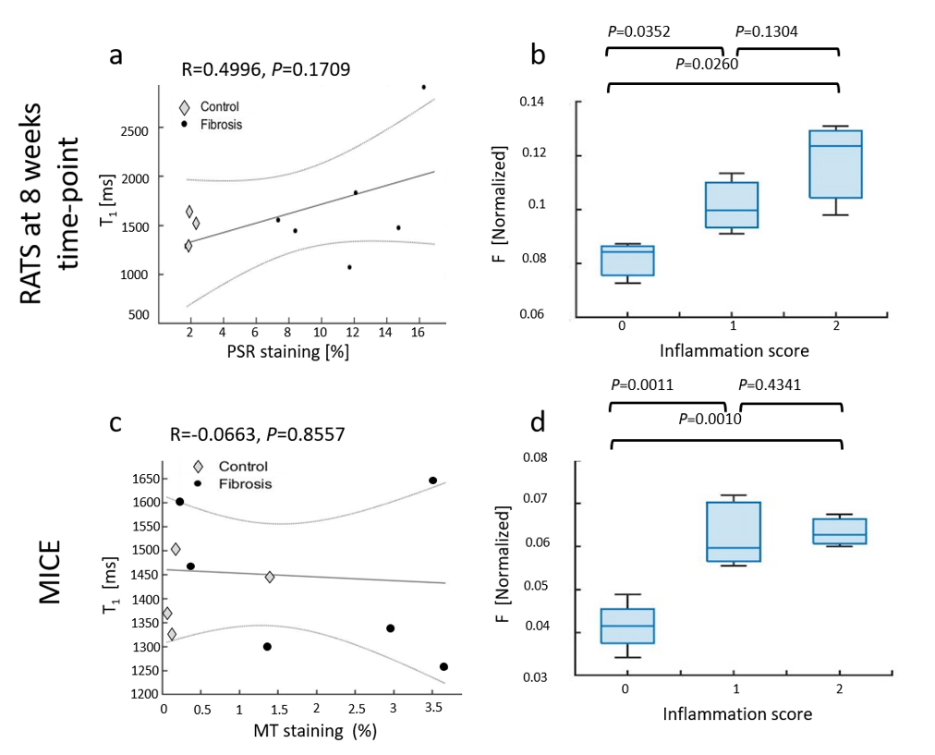


**Figure S1 –** Correlation charts between the histology staining percentage based on the spectral segmentation to F for rats after 8 weeks of injections **(a)** and mice **(c)**. A comparison between F value and inflammation score for rats at 8 weeks time-point **(b)** and mice **(d)**.

*Rat histogram analysis*

Based on the histograms of F maps of all animals at 6 weeks and 8 weeks the percentage of pixels in the range 0.1< F was calculated. Average histograms at 6 weeks and 8 weeks for both groups are presented in Fig. S1a-b,d-e, respectively. The percentage of pixels showed significant difference at between the groups at 6 weeks, but not significant at 8 weeks (*P*=0.6251).


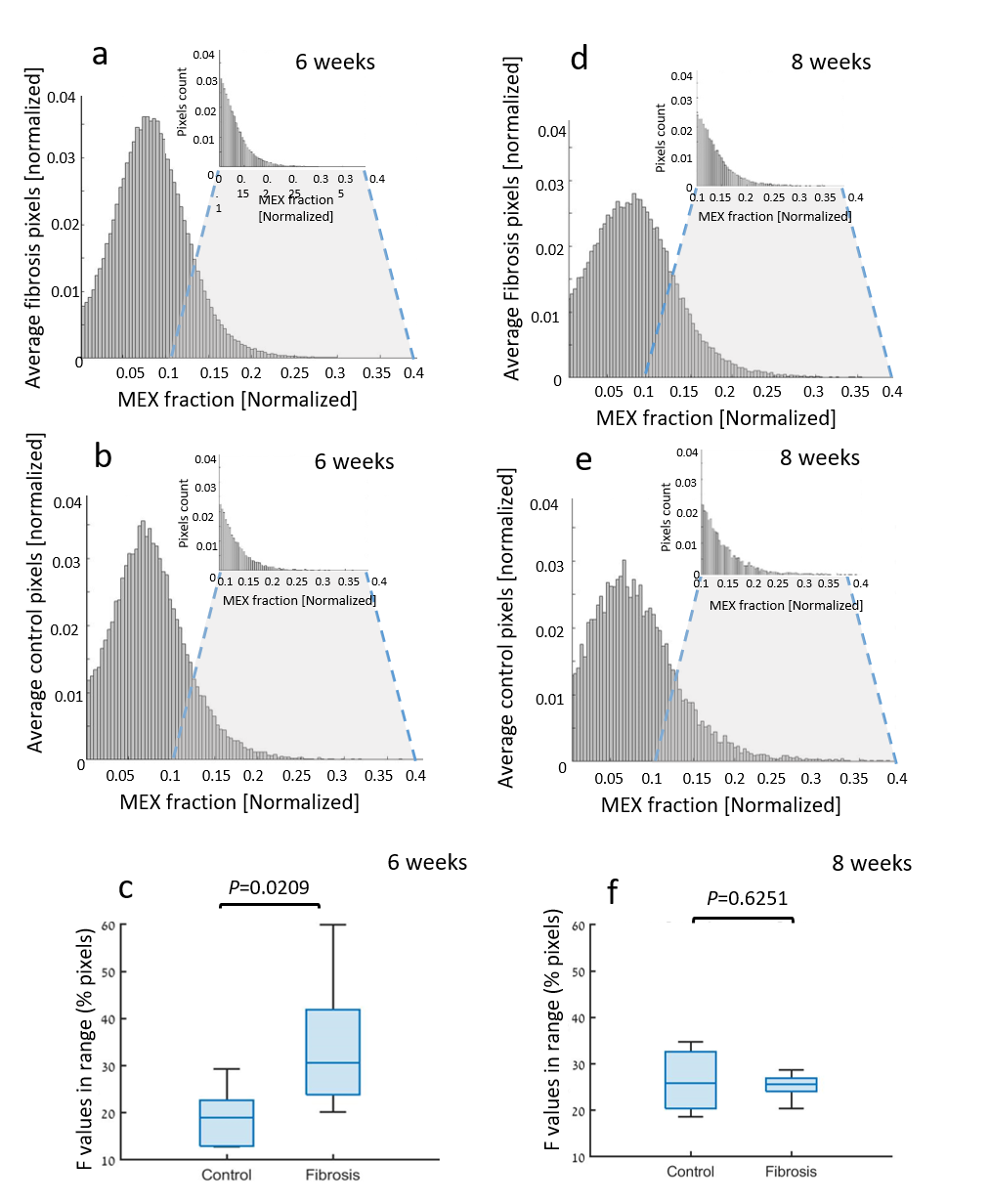


**Figure S2 -** Average histograms of F maps at 6 weeks time-point for fibrosis **(a)** and control **(b)** and 8 weeks time-point (**(d)** and **(e)**, respectively), normalized by total number of pixels. The insert is a zoomed-in display of the pixels in the range [0.1-0.4]. Based on the histograms of each animal, the percentage of pixels with F values in the upper range [0.1-0.4] was calculated and compared between CCl_4_ treated and control animals at 6 weeks **(c)** and 8 weeks **(f)**.
